# Interplay of Chemokines Receptors, Toll-like Receptors, and Host Immunological Pathways

**DOI:** 10.3390/biomedicines11092384

**Published:** 2023-08-25

**Authors:** Yuan-Tung Chu, Min-Tser Liao, Kuo-Wang Tsai, Kuo-Cheng Lu, Wan-Chung Hu

**Affiliations:** 1 Department of Anatomic Pathology, Taipei Tzu Chi Hospital, Buddhist Tzu Chi Medical Foundation, New Taipei City 231, Taiwan; chuyuantung@yahoo.com; 2 Department of Pediatrics, Taoyuan Armed Forces General Hospital Hsinchu Branch, Hsinchu 300, Taiwan; liaoped804h@yahoo.com.tw; 3 Department of Pediatrics, Taoyuan Armed Forces General Hospital, Taoyuan 325, Taiwan; 4 Department of Pediatrics, Tri-Service General Hospital, National Defense Medical Center, Taipei 114, Taiwan; 5 Department of Medical Research, Taipei Tzu Chi Hospital, Buddhist Tzu Chi Medical Foundation, New Taipei City 231, Taiwan; kwtsai6733@gmail.com (K.-W.T.); kuochenglu@gmail.com (K.-C.L.); 6 Division of Nephrology, Department of Medicine, Fu-Jen Catholic University Hospital, School of Medicine, Fu-Jen Catholic University, New Taipei City 242, Taiwan; 7 Department of Clinical Pathology, Taipei Tzu Chi Hospital, Buddhist Tzu Chi Medical Foundation, New Taipei City 231, Taiwan; 8 Department of Biotechnology, Ming Chuan University, Taoyuan 333, Taiwan

**Keywords:** chemokine receptors, Toll-like receptors, T helper cells

## Abstract

A comprehensive framework has been established for understanding immunological pathways, which can be categorized into eradicated and tolerable immune responses. Toll-like receptors (TLRs) are associated with specific immune responses. TH1 immunity is related to TLR7, TLR8, and TLR9, while TH2 immunity is associated with TLR1, TLR2, and TLR6. TH22 immunity is linked to TLR2, TLR4, and TLR5, and THαβ (Tr1) immunity is related to TLR3, TLR7, and TLR9. The chemokine receptor CXCR5 is a marker of follicular helper T cells, and other chemokine receptors can also be classified within a framework based on host immunological pathways. On the basis of a literature review on chemokines and immunological pathways, the following associations were identified: CCR5 with TH1 responses, CCR1 with TH1-like responses, CCR4 (basophils) and CCR3 (eosinophils) with TH2 and TH9 responses, CCR10 with TH22 responses, CCR6 with TH17 responses, CXCR3 with THαβ responses, CCR8 with regulatory T cells (Treg), and CCR2 with TH3 responses. These findings contribute to the identification of biomarkers for immune cells and provide insights into host immunological pathways. Understanding the chemokine and Toll-like receptor system is crucial for comprehending the function of the innate immune system, as well as adaptive immune responses.

## 1. Introduction of Host Immunological Pathways

The comprehensive and updated framework of host immune reactions can be categorized into two types: immunoglobulin G (IgG)-related eradicable immune responses and IgA-related tolerable immune reactions [1,2]. Eradicable immune reactions refer to the stronger host immune responses aimed at eliminating pathogens in the early stages. Contrarily, tolerable immune reactions are induced when diffuse organ infections, such as chronic HBV liver infection, occur. In such cases, host immune reactions tend to be milder to prevent severe organ damage or failure. The initiation of antibody isotype switch from IgM to IgG, facilitated by follicular helper T (Tfh) cells and influenced by the cytokine interleukin-(IL-)21, triggers the development of eradicable immune reactions [3]. There are four eradicable immunological pathways corresponding to different types of infectious pathogens. The TH1 immunologic response is the host immunity fighting intracellular bacteria, protozoa, and fungi [4]. This type of immunity is activated by the cytokine IL-12 and involves several immune cells, including type 1 macrophages (M1), CD4 T cells producing interferon-gamma (IFN-γ), cytotoxic T cells (group 1 effector-memory CD8 T cells), group 1 invariant natural killer T (iNKT1) cells, and IgG3-secreting B cells [5,6]. The TH1 immunological response plays a vital function in type 4 delayed-type autoimmunity. On the other hand, the TH2 immune reaction functions as the host’s immune response fighting parasites. TH2 immunity is further categorized to two kinds of sub-groups: TH2a and TH2b [7]. The cytokine IL-4 drives TH2 immunity. TH2a and TH2b immune responses serve as host immune mechanisms against endoparasites (helminths) and ectoparasites (insects), respectively. The TH2a immune response involves inflammatory eosinophils, CD4 T cells producing interleukin (IL)-4/5, mast cells with tryptase activity (MCt), iNKT2 cells, and IgG4 B cells. TH2b immunity includes basophils, CD4 T cells producing IL-13/4, mast cells with tryptase/chymase activity (MCtc), iNKT2 cells, and immunoglobulin E (IgE) B cells. TH2 immunity is associated with type 1 allergic hypersensitivity. TH22 immunity represents the host’s immunological reaction against extracellular bacteria, protozoa, and fungi. It involves neutrophils (N1), T helper cells secreting IL-22, iNKT17 cells, and IgG2 B cells [8,9]. TH22 immunity is associated with type 3 immune complex-mediated hypersensitivity and is driven by cytokines TNFα, IL-1, and IL-6. THαβ immunity is the host’s immune response against infectious molecules such as viruses and prions [10,11,12]. It involves type 1 NK cells (NK1), IL-10-secreting CD4 T cells, iNKT10 cells, CD8 T cells (Tc2 or EM1), and IgG1 B cells [13]. THαβ immunity is linked to type 2 antibody-dependent cytotoxic hypersensitivity and is driven by cytokines IFN-α or IFN-β. The initiation of THαβ immunity is also aided by IL-27 and IL-10.

**Figure 1:**
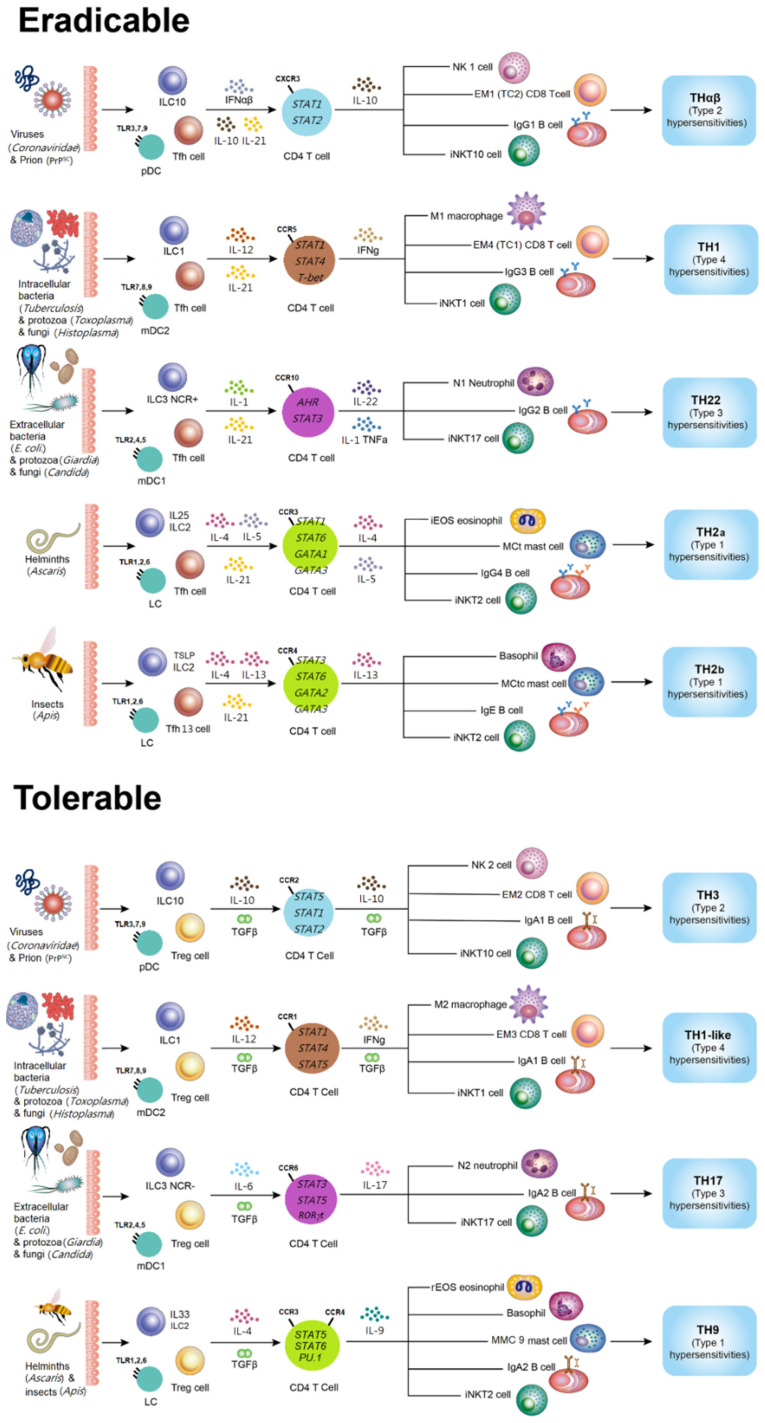
The framework of host immunological pathways. Basically, there are IgG-dominant eradicable immune responses and IgA-dominant tolerable immune responses. In eradicable immune reactions, there are TH1 anti-intracellular microorganism immunity, TH2a anti-helminth immunity, TH2b anti-insect immunity, TH22 anti-extracellular microorganism immunity, and THαβ anti-infectious molecule immunity. In tolerable immune reactions, there are TH1-like anti-intracellular microorganism immunity, TH9 anti-parasite immunity, TH17 anti-extracellular microorganism immunity, and TH3 anti-infectious molecule immunity. Abbreviations: ILC: innate lymphoid cell, mDC: myeloid dendritic cells, LC: Langerhans cell, IL: IL, EM: effector-memory, MC: mast cell, iNKT: invariant natural killer T cell, pDC: plasmacytoid dendritic cell, NK: natural killer cell, IFN: IFN, TGF: TGF, Treg: regulatory T cell, Tfh: follicular helper T cell, Tfh13: follicular helper 13 T cell, TLR: Toll-like receptor, CCR or CXCR: chemokine receptor. Adapted from W-C Hu. A framework of all discovered immunological pathways and their roles for four specific types of pathogens and hypersensitivities, Hu, W.C. 1992 [1].

Tolerable immune reactions can also be categorized into four types according to types of pathogens. These reactions are initiated by regulatory T cells, which induce a switch in antibody class to IgA [14,15,16]. Treg cells play a crucial role in promoting IgA class switch in Peyer’s patches of the intestine, thereby connecting mucosal immunity and oral tolerance. Oral tolerance can be induced to prevent harmful host immune reactions against food proteins. In addition, mucosal immunity with IgA secretion can help to mildly control co-existing bacteria in intestinal tracts. Thus, tolerable immunity is vital to mucosal immunity and oral tolerance. Tolerable immunity induced by Treg cells can help the physiologic function of both oral tolerance and mucosal immunity. Consequently, Treg cells are essential for tolerable immune responses. *FOX3P* serves as the key transcription factor regulating CD4 T cells, while transforming growth factor (TGF)-β functions as the key cytokine. The host’s tolerable immune response against intracellular bacteria, protozoa, and fungi is referred to as the TH1-like immune reaction. Furthermore, TH1-like cells that secrete FOX3P(+) IFNγ have been identified after stimulation by Treg plus TH1 condition [17,18]. Type 4 delayed-type hypersensitivity involves the following immune cells: M2 macrophages, CD4 T cells that produce TGF-β and IFNγ, iNKT1 cells, CD8 T cells (EM3), and IgA1 B cells [19,20]. This immune response is associated with the release of TGF-β and IL-12, which drive a TH1-like immune reaction. M2 macrophages can be stimulated by TGF-β plus TH1 cytokines. Another immune reaction called the TH9 immune reaction is the host’s tolerable immune physiology that fights against parasites: insects and helminths. It consists of regulatory eosinophils (rEOS), basophils, IL-9-secreting T helper cells, iNKT2 cells, mast cells (MMC9), and IgA2 B lymphocytes [21,22]. Type 1 allergic hypersensitivity is associated this immunity. The key cytokines involved in the immune response are IL-4 and TGF-β, which drive the TH9 immune reaction. TH17 immunity represents the host’s tolerable immune reaction against extracellular bacteria, protozoa, and fungi. This response involves immune cells, including neutrophils (N2), CD4 T helper cells that secrete IL-17, iNKT17 cells, and B lymphocytes producing IgA2. [23] The immune response mentioned is correlated with group 3 immune complex-mediated autoimmunity. The driver cytokines for the TH17 immune response are IL-6 and TGF-β. TH3 immunity serves as the host’s immune physiological mechanism to combat infectious molecules such as viruses and prions [12,24,25,26]. This immune reaction involves type-2 NK cells (NK2), CD4 T cells that secrete both IL-10 and TGF-β, iNKT10 cells, CD8 T cells (EM2), and B cells producing IgA1. It is associated with type 2 antibody-dependent cytotoxic hypersensitivity. The driver cytokines for the TH3 immune reaction are IL-10 and TGF-β. For a visual representation of the immunology pathways, please refer to Figure 1.

## 2. Introduction to Toll-Like Receptors

Toll-like receptors are evolutionarily conserved molecules that sense pathogens and initiate immune responses. Humans have 10 Toll-like receptors (TLRs), named TLR1 to TLR10. TLR10 is unlike other TLRs and is thought to be the immune regulator of Toll-like signaling [27,28]. Toll-like receptors (TLRs) play a crucial role in triggering both innate and adaptive immune responses. They not only initiate innate immune reactions but also serve as vital connectors between innate and adaptive immunity. TLRs are typically activated in a cooperative manner, which helps prevent false positive alarms and ensures the initiation of immune reactions specifically in response to real pathogen infections. The host immunological pathways can be categorized into four distinct types on the basis of the specific types of pathogens they respond to: viruses/prions, intracellular bacteria/protozoa, extracellular bacteria/fungi, and helminths/insects. In the upcoming sections, each TLR is discussed, highlighting its role in initiating these four types of host immunological pathways.

## 3. TLRs and TH1 Immunological Pathway

The TH1 host immune response specifically targets intracellular bacteria and protozoa. This immunological pathway relies on macrophages as the main effector cells. Additionally, macrophages play a crucial role as antigen-presenting cells in the host. It is important to note that the TH1 response is mounted against intracellular pathogens [1]. TLRs located in the endosomal compartments of macrophages play a vital role in initiating immune response signals. The key TLRs involved in initiating TH1 immune reactions are TLR7, TLR8, and TLR9 [29,30,31,32,33,34]. These TLRs are all situated in the endosomal compartments of macrophages. Macrophages phagocytize intracellular bacteria or protozoa, digesting them within the endolysosomes. Ligands for TLR7, TLR8, and TLR9 are single-stranded RNA, bacterial ribosomal RNA, and unmethylated CpG DNA oligonucleotides, respectively. These ligands are generated during the digestion of bacteria or protozoa in macrophage phagolysosomes. The cooperation between TLR7, TLR8, and TLR9 signaling facilitates the initiation of TH1 immune responses by triggering the production of IL-12. The major TLR7, TLR8, and TLR9 signaling pathways involve IRF5 and IRF7, which upregulate downstream targets such as IL-12 and IFN-gamma [35,36,37,38]. TLR8 signaling activates IRF5, whereas TLR7/9 signaling activates IRF7 [39]. The cooperative action of IRF5 and IRF7 leads to the production of the cytokine IL-12, which plays a role in the immune response against intracellular bacteria and protozoa. Additionally, TLR7/8/9 stimulation induces the upregulation of TH1-related cytokines, including IL-12 and IFN-γ, through cytokine induction.

TLR signaling that leads to TH1 immunity is discussed below. TLR7 and TLR9 first activate MyD88 complexes. These MyD88 complexes, termed myddosomes, also contain TRAF3, TRAF6, IRAK4, IRAK1, IKKα, OPNi, and DOCK2. The activated MyD88 activates IRAK1; subsequently, IRAK1 activates IKKα. Next, IKKα is translocated into the nucleus, where it triggers the transcription factor IRF7 [40]. IRF7 can also be phosphorylated by IRAK1 [39]. In addition, TLR8 first activates MyD88. In turn, MyD88 activates TAK1, which then activates IKKβ. TAK1 can also activate the downstream molecule JNK. When IKKβ is translocated into the cell nucleus, it triggers the transcription factor IRF5. TLR7, TLR8, and TLR9 can interact with the TLR adaptor TASL to trigger the downstream transcription factor IRF5. IRF5 can trigger IL-12 and IFNγ production to activate the TH1 immunological pathway [35].

Autoimmune disorders involving the TH1 immunological pathway include type 1 diabetes, multiple sclerosis, contact dermatitis, and tuberculosis. Previous studies have revealed that TLR7, TLR8, and TLR9 are associated with these TH1-dominated autoimmune illnesses.

## 4. TLRs and TH2 Immunological Pathway

The TH2 immunological pathway is responsible for the host’s immunity against helminths and insects. Eosinophils, basophils, and mast cells are the main effector cells involved in the host’s immune response mediated by TH2 [7]. TLR1, TLR2, and TLR6 are the major Toll-like receptors (TLRs) involved in the TH2 immunological pathway [41,42,43,44,45,46]. TLR1, TLR2, and TLR6 are surface receptors found on antigen-presenting cells such as basophils or mast cells. TLR2 can form heterodimers with either TLR6 or TLR1 on the cell surface. Studies using animals with TLR2 knockout have shown that TLR2 is involved in the development of asthma and is associated with the TH2 immunological pathway. Since helminths and insects reside outside host cells, Toll-like receptors that respond to these pathogens are expected to be located on the cell surface. As TLR2 and TLR6 are present on the cell surface, they initiate TH2 immune responses. The specific ligand for the TLR2–TLR6 complex is diacyl lipopeptide [45,47]. Chitin, the primary component of the insect exoskeleton, functions as a ligand for TLR2 [48,49]. TLR2 plays a crucial role in TH2 immunity and is associated with the pathophysiology of helminth infections. TLR1 is also involved in initiating TH2 immune reactions. TLR2 forms heterodimers with TLR1 and TLR6; together, they recognize triacyl lipoproteins. The signaling pathways of TLR1, TLR2, and TLR6 involve TIRAP (MAL) adaptor molecules, which activate a MyD88-independent pathway that triggers the TH2 immune response, including the production of IL-4 cytokines. Additionally, TLR2–TLR6 heterodimers activate the PI3K and AKT pathway, which promotes the progression of the TH2 immune response. TLR2 activation leads to the activation of TPL2 (MAP3K8), which in turn activates IKK2, resulting in the activation of NFκB. TPL2 also activates MEK1/2 and subsequently ERK1/2, which trigger the TH2 immunological pathway. The RAC1/PI3K/AKT pathway also activates ERK1/2 signaling, contributing to TH2 immunity [50,51].

TH2-dominated hypersensitivity is closely associated with allergic diseases related to type 1 IgE, such as asthma, allergic rhinitis, and atopic dermatitis [43]. The upregulation of TLR1, TLR2, and TLR6 is correlated with TH2-dominated allergic diseases. These findings indicate that TLR1, TLR2, and TLR6 play critical roles in the development of these allergic disorders. Additionally, upon stimulation of TLR1/2/6, TH2-related cytokines such as IL-4, IL-25, and IL-33 are upregulated.

## 5. TLRs and TH22 Immunological Pathway

TH22 immunity copes with the infections of extracellular protozoa, bacteria, and fungi. Neutrophils, IL-22-secreting CD4 T cells, iNKT17 cells, and IgG2 B cells are the major effector cells involved in the TH22 immune response. The main Toll-like receptors (TLRs) associated with the TH22 immunological pathway are TLR2, TLR4, and TLR5 [52,53,54,55]. TLR2 homodimers recognize peptidoglycans and lipoteichoic acid in the cell walls of Gram-positive bacteria, while also interacting with beta-glucans in fungal cell walls. TLR4 is responsible for recognizing LPS endotoxins in the cell walls of Gram-negative bacteria. TLR5, on the other hand, recognizes flagellin found in motile bacteria. These molecules play crucial roles in defending against extracellular bacteria and fungi. Neutrophils, dendritic cells, and monocytes/macrophages express TLR2, TLR4, and TLR5 on their cell surfaces. The classical MyD88 signaling pathway is activated by TLR2, TLR4, and TLR5, leading to the production of proinflammatory cytokines such as IL-1, IL-6, and TNF-α. This pathway activates NFκB and AP1, which also contribute to the activation of the TH22 immunological pathway.

TLR4 signaling triggers the formation of myddosomes, which consist of MyD88, IRAK1, and IRAK4. IRAK4 activates IRAK1, leading to the autophosphorylation of IRAK1 and its dissociation from the MyD88 complex [47,56]. Subsequently, IRAK1, in association with the ubiquitin-conjugating enzymes UBC13 and UEV1A, facilitates K63-linked polyubiquitination of TRAF6 and TAK1 complexes. Activation of IRAK1 induces TAK1 activation, followed by TRAF6 activation. TAK1, a member of the MAPKKK family, forms a complex with regulatory subunits TAB1, TAB2, and TAB3. TAB1, TAB2, and TAB3 interact with the polyubiquitin chain of TRAF6 to promote TAK1 activation. TAK1 activation leads to the activation of the IKK complex, NFκB, and MAPKs. The IKK complex comprises catalytic subunits IKKa and IKKb, along with the regulatory subunit IKKg. TAK1 binds to the entire IKK complex, and then phosphorylates and activates IKKb. The IKK complex then phosphorylates the NFκB inhibitor protein IKBa. Subsequently, IKBa undergoes proteasomal degradation, resulting in the translocation of NFκB into the nucleus, which triggers the expression of inflammatory genes. Furthermore, TAK1 activation also leads to MAPK activation, and MAPK, through the p38 (Fos)-MKK3/6 and the JNK (Jun)-MKK7 pathways, activates the transcription factor AP1 [57,58,59,60,61].

The TH22-dominated immunological pathway is associated with type 3 immune-complex hypersensitivity, which includes conditions such as Arthus reactions and rheumatoid arthritis [52,55,62]. In autoimmune diseases characterized by TH22-/TH17-dominated immune responses, such as rheumatoid arthritis and Arthus reactions, the expression of TLR2, TLR4, and TLR5 is upregulated. Stimulation of TLR2/4/5 induces the upregulation of pro-inflammatory cytokines related to TH17/TH22, including TNF-α, IL-1, IL-6, and IL-8.

## 6. TLRs and THαβ Immunological Pathway

The THαβ immunological pathway is responsible for the host’s immune response against viral infections. It engages several key effector cells, such as NK cells (antibody-dependent cellular cytotoxicity), CD8 T cells, iNKT10 cells, and IgG1 B cells. In the context of the THαβ immune response, Toll-like receptors (TLRs) play a significant role, particularly TLR3, TLR7, and TLR9 [63,64,65,66]. TLR3 recognizes double-stranded RNA (dsRNA) produced during viral replication, while TLR7 detects single-stranded RNA (ssRNA) also present during this stage. TLR9, on the other hand, recognizes unmethylated CpG DNA found in both bacterial and viral genomes. These receptors are primarily expressed in the endosomal compartment of plasmacytoid dendritic cells, which serve as crucial antigen-presenting cells during viral infections. The endosomal compartment provides an ideal setting for the interaction between viruses and TLRs, given that viruses are intracellular pathogens. Upon activation, TLR3 triggers the TRIF–IRF3 signaling pathway, while TLR7 and TLR9 activate the IRAK1–IRF7 signaling pathway. Activation of IRF3 and IRF7 leads to the production of type 1 interferons (IFNs), which play a vital role in mounting an effective antiviral immune response. In summary, the signaling pathways involving TLR3, TLR7, and TLR9 converge on IRF3 and IRF7, initiating the downstream type 1 IFN signaling pathway to initiate the THαβ immune response.

The THαβ immune response involves specific signaling pathways mediated by Toll-like receptors (TLRs). The TLR3 signaling pathway directly activates TRIF, which subsequently triggers a cascade involving TRAF3, TBK1, and IKKε [56]. Upon translocation into the cell nucleus, IKKε activates the transcription factor IRF3. On the other hand, TLR7 and TLR9 activate the MyD88 pathway. MyD88 then activates IRAK1, which further activates IKKα. When IKKα translocates into the nucleus, it activates the transcription factor IRF7. Both IRF3 and IRF7 have the capability to activate the production of IFN-α and IFN-β, thereby initiating the THαβ immune response [40,67].

**Figure 2:**
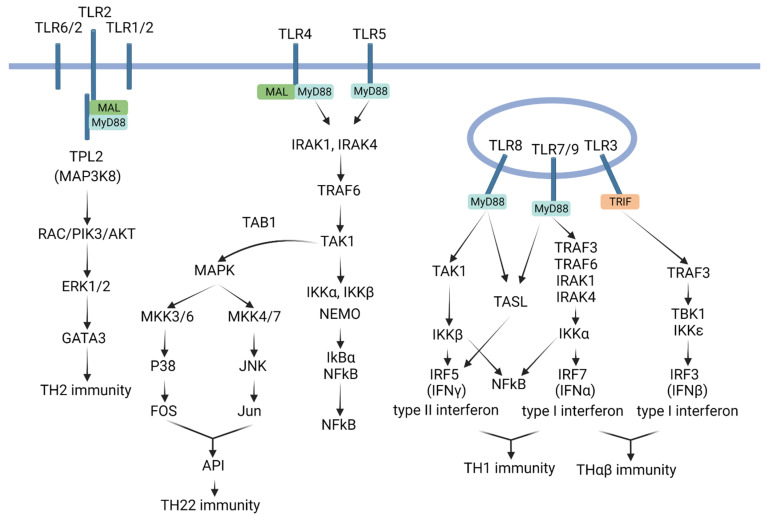
TLRs in the framework of immunological pathways. TH1 immunity is related to TLR7, TLR8, and TLR9. TH2 immunity is related to TLR1, TLR2, and TLR6. TH22 immunity is related to TLR2, TLR4, and TLR5. THαβ immunity is related to TLR3, TLR7, and TLR9.

Autoimmune diseases that involve the THαβ immunological pathway are characterized by type 2 antibody-dependent cellular cytotoxic hypersensitivity reactions. In this category, typical illnesses such as Graves’ disease, myasthenia gravis, and systemic lupus erythematosus [63,68,69,70] are observed. Upregulation of TLR3, TLR7, and TLR9 is observed in this type of hypersensitivity. Upon stimulation by TLR3/7/9, cytokines associated with the THαβ pathway, including type 1 interferons, are upregulated. A summary of the associations between TLRs and the framework of host immunological reactions can be seen in Figure 2.

## 7. TLRs and Their Implications

TLR agonists have been developed to trigger the host immune response against cancer by stimulating TLRs. One example is Coley’s toxin, a BCG mixture used as an adjuvant in bladder cancer treatment, which can upregulate TLR2 and TLR4. Researchers are also studying other TLRs, including TLR3, TLR7, and TLR9, as potential treatments for cancer. These TLRs have the potential to induce apoptosis in cancer cells, similar to the destruction of cells infected by viruses. Utilizing oncolytic viruses, we can stimulate the immune response of the host to combat cancer cells effectively. Beyond this, Toll-like receptors (TLRs) play a significant role in vaccine development. To enhance vaccine efficacy against parasites, TLR1, TLR2, and TLR6 agonists can be employed. Alternatively, vaccines targeting viruses and prions can be bolstered by TLR3, TLR7, and TLR9 agonists. When addressing intracellular microorganisms such as tuberculosis, TLR7, TLR8, and TLR9 agonists hold promise for vaccine formulation. For extracellular microorganisms such as enterohemorrhagic E. coli, the use of TLR2, TLR4, and TLR5 agonists is applicable. Expanding the spectrum of TLR applications, they also show potential in treating autoimmune diseases. Specifically, in cases of THαβ-related autoimmune conditions such as systemic lupus erythematosus, TLR3, TLR7, and TLR9 antagonists can be used to mitigate hypersensitivity reactions. Similarly, for TH2-associated autoimmune ailments such as asthma or atopic dermatitis, TLR1, TLR2, and TLR6 antagonists offer treatment avenues. Addressing TH1-associated autoimmune disorders such as type 1 diabetes, TLR7, TLR8, and TLR9 antagonists present therapeutic possibilities. Moreover, for TH22/17-associated autoimmune conditions including rheumatoid arthritis, TLR2, TLR4, and TLR5 modulation holds potential for managing these disorders. These ongoing investigations in this dynamic field hold considerable promise [71,72,73,74,75,76,77,78,79].

## 8. Introduction to Chemokine Receptors

The chemokine system plays a crucial role in attracting immune cells to sites of tissue damage and in facilitating the accumulation of immune cells in immune system organs such as the bone marrow, thymus, and lymph nodes. The initiation of an effective immune response is triggered by follicular helper T (Tfh) cells, and the chemokine receptor CXCR5 serves as a biomarker for these cells [3]. CXCR5 promotes the migration of Tfh cells to the germinal center, where they assist B cells in producing IgG antibodies. This report provides a comprehensive review of the literature on the roles of chemokine receptors in modulating other immune cells, establishing a framework for understanding host immunological pathways [1].

## 9. TH1 Immunological Pathway and C–C Motif Chemokine Receptor (CCR) 5

The TH1 host immunological pathway is an immune response mounted by the host against intracellular bacteria, fungi, and protozoa. This pathway is driven by the cytokine IL-12, and the key transcription factors involved in mediating TH1 immunity are STAT1 and STAT4 [7]. The effector cells associated with the TH1 immune response include M1 macrophages, CD4 T cells that secrete IFN-γ, CD8 T cells (specifically CD28+ CD27− Tc1 EM4 cells), invariant natural killer T1 (iNKT1) cells, and IgG3 B cells [19]. There are two chemokine receptors associated with traditional TH1 immunity: CCR5 and CXCR3 [20,80,81]. The traditional TH1 immune response can be categorized into two types: TH1 immune response against intracellular bacteria and protozoa, and THαβ immune response against viruses. Studies suggest that CCR5 plays a crucial role in TH1 immunity against intracellular bacteria and protozoa. CCR5 is expressed on TH1 CD4 lymphocytes and monocytes/macrophages, which are major effector cells involved in TH1 immunity. In type 1 cytotoxic T cells, CCR5 is the predominant chemokine receptor expressed. However, CCR5 is also expressed in iNKT1 cells. The ligands of CCR5 include C–C motif chemokine ligands (CCL) 3 and CCL4, also known as macrophage inflammatory protein (MIP) 1α and 1β, respectively. These findings support that CCR5 is a key chemokine receptor in the TH1 immunological pathway.

## 10. TH2/TH9 Immunological Pathway and CCR4/CCR3

The TH2 immunological pathway is the host’s immune response against helminths and insects. The cytokine responsible for driving the TH2 immune reaction is IL-4. In TH2 immunity, STAT6 has been identified as the key transcription factor [7]. The TH2 immune response involves effector cells such as stimulating eosinophils, basophils, mast cells, CD4 T cells that secrete IL-4/IL-5, iNKT2 cells, and B cells producing IgG4/IgE. These cells play a role in combating helminths and insects. On the other hand, the TH9 immunological pathway is associated with the host’s tolerable immune response against helminths and insects. The TH9 immune reaction is driven by the cytokines IL-4 and TGF-β. STAT5 and STAT6 are the key transcription factors involved in TH9 immunity. In the TH9 immune response, regulatory eosinophils, basophils, mast cells (MMC9), CD4 T cells that secrete IL-9, iNKT2 cells, and B cells producing IgA2 are present. The chemokine receptors CCR4 and CCR3 are associated with both TH2 and TH9 immunity [82,83]. CCR4 is expressed on the surface of type 2 IL-4-secreting CD4 T cells, activated basophils, and MCt cells [84]. Additionally, iNKT2 cells also express CCR4 on their surface. The ligands of CCR4 are CCL17 (TARC) and CCL22 (MDC) [85]. CCR3 can be found on IL-9-secreting CD4 T cells, activated eosinophils, MCct cells, and iNKT2 cells. The ligands of CCR3 are eotaxin (CCL11) and eotaxin-3 (CCL26).

## 11. TH22 Immunological Pathway and CCR10

The TH22 immunological pathway plays a crucial role in the host’s immune response against extracellular bacteria, protozoa, and fungi. This immune response is driven by the cytokines IL-1 and tumor necrosis factor-α [8,9]. The key transcription factor associated with TH22 immunity is STAT3. Effector cells involved in the TH22 immune response consist of neutrophils (N1s), CD4 T cells that secrete IL-22, iNKT17 cells, and B cells producing IgG2. The chemokine receptor associated with the TH22 immune reaction is CCR10 [8,9,86]. CCR10 is expressed on the surface of IL-22-secreting CD4 T cells [86]. These cells are skin-homing T cells that provide defense against extracellular bacteria. The first line of defense against extracellular bacteria and fungi is the epithelial barrier. CCR10 ligands, such as CCL27 (CTACK) and CCL28 (MEC), are involved in this process. Additionally, CD4 T cells that secrete IL-8 play a role in TH22 immunity. Hence, the chemokine receptors CXCR1/CXCR2 (IL-8 chemokine receptors) could be significant in both TH22 immunity and innate immunity. CXCR1 is associated with N1 neutrophils, while CXCR2 is related to N2 neutrophils (tumor-associated neutrophils).

## 12. THαβ Immunological Pathway and CXCR3

The THαβ immunological pathway is an immune response pathway employed by the host to defend against viruses and prions. This pathway involves the activation of cytokines such as IFN-α/β and IL-10. The key transcription factors associated with THαβ immunity are STAT1 and STAT2. The effector cells involved in the THαβ immune response include NK cells (NK1), IL-10-secreting CD4 T cells (also known as type 1 regulatory cells [Tr1]), CD8 T cells (CD27+ CD28+ TC2, EM1), iNKT10 cells, and IgG1 B cells [19]. CD27 is a protein expressed in CD8 T cells that is known to be associated with antiviral immunity. The chemokine receptor CXCR3, which is related to the THαβ pathway, plays a key role in traditional TH1 immunity [87,88,89]. While CXCR3 expression is commonly observed in CD4 T cells, it is also detected in NK cells and CD8 T cells, aiding in the defense against viral infections. CXCR3 can bind to three IFN-inducible ligands: CXCL9 (MIG), CXCL10 (IP-10), and CXCL11 (I-TAC) [87,89,90]. Type 1 IFNs can upregulate these chemokines during viral infection.

## 13. Regulatory T Cells (Tregs) and CCR8

Regulatory T cells (Tregs) play crucial roles in triggering tolerable immune responses. Tregs are characterized by the expression of CD25 and CD4, and they secrete transforming growth factor (TGF)-β. In addition to Tregs, other cell types such as regulatory dendritic cells, regulatory B cells, CD8 Tregs, and regulatory iNKT cells also contribute significantly to this category of immune response. The master chemokine receptor associated with Tregs is CCR8 [14,91]. CC chemokine receptor 8 (CCR8) plays a crucial role in the functions of TGF-β-secreting regulatory T cells (Tregs), and its ligand is CCL1. It is worth noting that Tregs may also express other chemokine receptors to co-localize with other effector cells within the host’s immunological pathway, thereby facilitating their regulatory functions.

## 14. TH1-like Immunological Pathway and CCR1

The TH1-like pathway within the host’s immunological response is an effective immune defense against intracellular bacteria and protozoa. This immune response is characterized by the production of cytokines such as IL-12 and TGF-β [1]. The transcription factors STAT5, STAT1, and STAT4 play pivotal roles in TH1-like immunity. Effector cells involved in the TH1-like immune response include M2 macrophages, CD4 T cells producing IFN-γ and TGF-β, CD8 T cells (CD28- CD27- EM3), iNKT1 cells, and IgA1 B cells. The primary chemokine receptor associated with TH1-like immune reactions is CCR1 [92]. The ligand for CCR1 is monocyte chemoattractant protein-6 (CCL6) and CCL9. CCR1 can be upregulated in M2 macrophages [20,93]. Chemokines, specifically CCL6 and CCL9, primarily attract monocytes/macrophages. The chemokine receptor CCR1 is mainly expressed on the surfaces of monocytes/macrophages and TH1-like CD4 T cells.

## 15. TH17 Immunological Pathway and CCR6

The TH17 immunological pathway is the host’s tolerable immune response against extracellular bacteria and fungi. The cytokines driven by TH17 immunity are IL-6 and TGF-β [1]. The key transcription factors of TH17 immunity are STAT5 and STAT3, and the effector cells of the TH17 immune response include neutrophils (N2), IL-17-secreting CD4 T cells, iNKT17 cells, and IgA2 B cells. The chemokine receptor related to the TH17 immune reaction is CCR6 [94]. CCR6 can be expressed on the surface of IL-17-secreting CD4 T cells. The ligand of CCR6 is CCL20 (MIP-3α).

## 16. TH3 Immunological Pathway and CCR2

The TH3 immunological pathway serves as the host’s tolerable immune response against viruses and prions. This pathway involves the production of specific cytokines, namely, TGF-β and IL-10. Key transcription factors associated with TH3 immunity include STAT1, STAT2, and STAT5. Effector cells involved in the TH3 immune response include NK cells (NK2), IL-10/TGF-β-secreting CD4 T cells, CD8 T cells (CD27+ CD28− TC2, EM2), iNKT10 cells, and IgA1 B cells. The chemokine receptor associated with the TH3 immunological pathway is CCR2 [95,96,97,98]. The chemokine receptor CCR2 has two ligands, namely, CCL2 (also known as MCP-1) and CCL12 (also known as MCP-5) [86,90]. The CCL2/CCR2 interaction can be upregulated by TGF-β or type 1 interferons to mediate the TH3 immune reaction [97,98].

## 17. Other Chemokine Receptors and Their Functions

**Figure 3:**
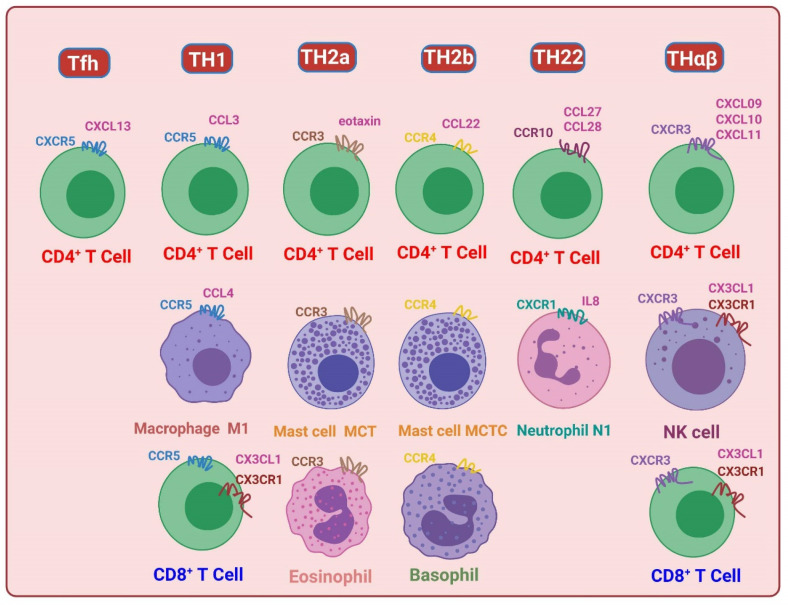
Chemokine receptors in the eradicable immunities. Tfh is related to CXCR5. TH1 response is related to CCR5, while TH2b and TH2a responses are related to CCR4 (basophils) and CCR3 (eosinophils), respectively. TH22 response is related to CCR10, and Thαβ response is related to CXCR3.

**Figure 4:**
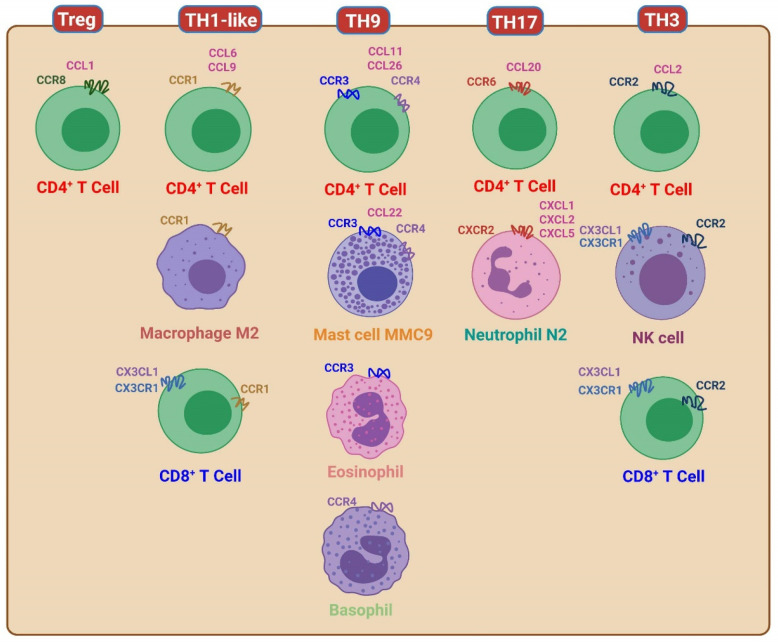
Chemokine receptors in the tolerable immunities. TH1-like response is related to CCR1, and TH9 response is related to CCR4 (basophils) and CCR3 (eosinophils). TH17 response is related to CCR6, regulatory T cells (Treg) are related to CCR8, and TH3 response is related to CCR2.

Chemokine receptors play crucial roles in immune cell migration and positioning. In addition to the previously mentioned chemokine receptors, there are other receptors that have important functions in immune cell homing. CCR7 is involved in immune cell homing into the T cell area of lymph nodes, facilitating the interaction between T cells and antigen-presenting cells [99]. CXCR5, on the other hand, is responsible for immune cell homing into the B cell area of lymph nodes, promoting interactions between B cells and other immune cells [3]. Furthermore, CCR9 plays a role in immune cell homing to the thymus, an important organ for T cell development [100,101]. CXCR1 and CXCR2 are chemokine receptors that play crucial roles in neutrophil chemotaxis and innate immunity. CXCR1 is specifically associated with N1 neutrophils, while CXCR2 is associated with N2 neutrophils. These receptors are expressed on various immune and non-immune cells, including neutrophils, macrophages, and endothelial cells [102,103]. Neutrophils are frontline cells of the immune system and are equipped with potent antimicrobial mechanisms. Proper recruitment of neutrophils is essential for combating microbial infections, maintaining homeostasis, regulating inflammation, promoting wound healing, and facilitating tissue repair. Chemokines, a class of signaling molecules, are responsible for orchestrating the precise migration of neutrophils. CXCR1 and CXCR2 recognize specific chemokines, including CXCL8, which belongs to the ELR+ CXC subfamily. CXCL8 is an important chemokine that targets neutrophils and activates them [102,103]. In addition to CXCR1 and CXCR2, there are other chemokine receptors involved in immune cell homing to specific organs. CXCR4 is implicated in immune cell homing to the bone marrow, which is vital for hematopoiesis and the maintenance of the hematopoietic stem-cell niche. CXCR6, on the other hand, plays a role in immune cell homing to the spleen, an important secondary lymphoid organ [100,104]. CX3CR1 is linked to NK cells and T cells, and XCR1 is used for dendritic cells for cross presentation to CD8 T cells [84,105,106]. A summary of chemokine receptors and their associations with the framework of host immunological pathways are shown in Figure 3 and Figure 4.

## 18. Chemokine Receptors and Their Implications

The information presented can be effectively applied to the treatment of autoimmune diseases. To address TH1/TH1-like autoimmune conditions, which include multiple sclerosis, employing CCR5 or CCR1 antagonists emerges as a potential therapeutic approach. In the case of TH2/TH9 autoimmune disorders such as bronchogenic asthma, the use of CCR3 or CCR4 antagonists presents a viable treatment strategy. Likewise, for TH22/TH17 autoimmune ailments such as rheumatoid arthritis, the application of CCR10 or CCR6 antagonists offers promise in disease management. THαβ/TH3 autoimmune diseases, such as systemic lupus erythematosus or sicca syndrome, could potentially be treated with CXCR3 or CCR2 antagonists. Furthermore, this knowledge holds relevance in the realm of cancer treatment. CCR1 antagonists can play a pivotal role in combating cancer cells by suppressing M2 tumor-associated macrophages, which express CCR1. This interaction presents an avenue to impede tumor growth. These implications collectively highlight potential avenues for future therapeutic interventions [107,108,109,110].

## 19. Conclusions

Improving our understanding of chemokine receptors and their relationships with various immunological pathways is crucial for the development of effective therapeutic strategies to treat immune-related diseases. In the context of immune-related diseases, such as rheumatoid arthritis, targeting specific chemokine receptors has been explored. Antibodies against CCR1, CCR2, and CCR5 have been developed for the treatment of rheumatoid arthritis. However, clinical trials have not shown efficacy for these treatments. Since rheumatoid arthritis is considered a TH17/TH22-related autoimmune disorder, this report suggests that CCR6 or CCR10 should be considered as potential drug targets. The findings mentioned above have significant implications for understanding host–pathogen interactions and the development of vaccines. By elucidating the roles of specific chemokine receptors in immune responses, researchers can pave the way for the development of targeted therapies and the design of future vaccines. To gain a deeper understanding of chemokine receptors and their potential as therapeutic targets, the reader can refer to the provided reference. This review article provides insights into the latest updates on chemokine receptors as therapeutic targets, including their mechanisms of action and potential strategies for future targeted therapies. Additionally, exploring the broader literature on cytokines, chemokines, and immune-mediated inflammatory diseases can further enhance knowledge in this field.

## Data Availability

Not applicable.
